# Implications to payers of switch from hospital-based intravenous immunoglobulin to home-based subcutaneous immunoglobulin therapy in patients with primary and secondary immunodeficiencies in Canada

**DOI:** 10.1186/1710-1492-10-23

**Published:** 2014-05-07

**Authors:** William C Gerth, Stephen D Betschel, Arthur S Zbrozek

**Affiliations:** 1 W. C. Gerth & Associates, LLC, 33 E End Avenue, Shrewsbury, New Jersey 07702, USA; 2 St. Michael's Hospital and the University of Toronto, Toronto, Ontario M5B 1 W8, Canada; 3 CSL Behring, King of Prussia, Pennsylvania 19406, USA

**Keywords:** IVIg, SCIg, Labor costs, Full time equivalents, FTEs, PID, SID, Nurse time

## Abstract

**Background:**

Switching primary/secondary immunodeficiency (PID/SID) patients from intravenous immunoglobulin (IVIg) to home-based subcutaneous immunoglobulin (SCIg) therapy reduces nurse time. A nurse shortage in Canada provides an important context to estimate the net economic benefit, the number of patients needed to switch to SCIg to recoup one full-time equivalent (FTE), and potential population-wide savings of reduced nurse time to a payer.

**Methods:**

The net economic benefit was estimated by multiplying the hourly compensation for nurses in Canada by the hours required for each administration route. The number needed to switch to SCIg to gain one nurse FTE was estimated by dividing the work hours in a year by the average annual savings in nursing time in a PID population in Canada. The prevalence of treated PID/SID in Canada was calculated using provincial IgG audit data to extrapolate the potential population-wide savings of switching patients to SCIg therapy.

**Findings:**

The net economic gain from switching one patient to home-based SCIg care would be C$2,603 (Canadian Dollars) in year 1 and C$2,948 each year thereafter. Switching 37 IVIg patients to SCIg would gain one nurse FTE. Switching 50% of the estimated 5,486 PID and SID patients in Canada receiving IVIg therapy to SCIg has the potential to save 223.3 nurse FTEs (C$23.2 million in labor costs).

**Conclusions:**

A shift from IVIg to less labor-intensive SCIg has the potential to help alleviate nurse shortages and reduce overall health care costs in Canada. Health care professionals might consider advocating for home-based SCIg therapy for PID/SID patients when clinically appropriate.

## Background

Canada has a shortage of registered nurses (RNs), predicted to increase to almost 60,000 full-time equivalents (FTEs) by 2022. [1] Professionals in the field suggest the national demand for nurses is masked by delayed retirements and heavy workloads. [2] Public-sector health-care nurses worked 21.54 million hours of paid and unpaid overtime in 2012 equal to 11,900 FTE jobs at a cost of C$925.5 million annually [3]. Strong links have been found in Canada between medication errors and the nurses’ workplace environment including working overtime, role overload, and perceived staffing shortages [4]. Increasing RN productivity by one percent per year would reduce the nurse shortage by nearly 50% by 2022 [1] and has the potential to cut nurse overtime.

Demand for hospital and office-based intravenous infusion therapy will grow with an aging demographic. Canadian Blood Services (CBS) reports 8.3% annual growth in overall immune globulin usage from 2006/07 to 2010/2011 [5]. In British Columbia, the number of patients receiving IVIg grew by 42.6% from 2003/04 to 2008/09 with PID/SID comprising 39.3% of total usage [6]. Infusion therapy is required in many other medical conditions including cancer, gastrointestinal diseases, and diseases requiring specialty infusion therapies like congestive heart failure and rheumatoid arthritis. Capacity to provide care to these patients will need to increase in terms of facilities and staffing.

SCIg replacement provides acceptable immunoglobulin (IgG) trough levels, a low incidence of side effects, similar efficacy to IVIg infusions, and faster functional recovery with less time off work in the treatment of PID [7]. There is evidence from North America that health-related quality of life and treatment satisfaction are improved when patients receiving IVIg are switched to home-based SCIg [8].

A recent report suggests that a key to slowing the growth of health spending is unlocking innovation to reduce the labor-intensity of care [9]. Home-based SCIg appears to provide a less labor-intensive means of care than hospital/office-based IVIg. In one Canadian study by Ducruet et al., the time spent by nurses in a PID pediatric center was 52.5 hours less per year with SCIg relative to IVIg, resulting primarily from a shift in watching over patients receiving IVIg to teaching patients to administer their SCIg. First year costs related to the nurse, technician and administration time were C$1,594 lower in patients switched from IVIg to SCIg (33.2% of overall C$4,801 savings with SCIg) [10]. In another Canadian study of adult PID by Martin et al., a switch from IVIg to home-based rapid push SCIg resulted in 45.2 fewer hours of nursing time in the first year post-switch and 51.2 fewer hours annually thereafter. Healthcare system costs were reduced by C$5,735 per patient treated with SCIg over three years compared to IVIg, principally because of less use of hospital nursing personnel [11]. These studies did not explore the trade-offs and variability payers across Canada may face in the efficient allocation of nurse staff around delivery of IgG to PID/SID patients.

The aim of this paper is to estimate the economic advantages from a payer perspective in Canada specifically from reduced nurse time patients with PID/SID who switch from hospital-based IVIg to home-based SCIg therapy.

## Methods

Three modeling approaches were taken to estimate the economic benefits of reduced nurse time from switching PID/SID patients from IVIg to SCIg: assessing the net economic benefit on short-term demands of nurse training to safely facilitate a switch; assessing the number of PID/SID patients needed to switch to gain one nurse FTE; and, assessing the population-wide savings potential across Canada.

### Net economic benefit

The cost in nursing time to educate and monitor the switching patients receiving IVIg to SCIg, and the savings from that expenditure, was estimated by multiplying the number of nursing hours and related labor costs in year 1 and in subsequent years for each route of administration. Nurse time estimates from Martin et al., of 45.2 and 51.2 fewer hours of nurse time in the first and annually in subsequent years post-switch to SCIg, were used in the base-case analysis. SCIg administration required 12 hours of RN time in year 1 (training 6, follow-up visits 6 hours) and 6 hours of RN time for follow-up visits in each subsequent year. IVIg required 57.2 hours of RN time each year assuming 14.3 visits per patient annually (two-thirds 13, one-third 17 visits) and a 4-hour infusion session each visit.

Hourly nurse compensation (C$57.58) was estimated by dividing the average annual wages and benefits of C$104,400 for one full time general duty nurse in British Columbia in 2011 [12] by the number of hours in a work year. The hours worked in a typical year was set at 1,813 hours (37-hour workweek; 49 work weeks) [3].

Hourly nurse compensation was multiplied by the hours required for each route of administration to estimate the net economic benefit in switching to SCIg. Estimates are provided on a per patient switched basis and per the number of patients needed to switch to recoup one nurse FTE.

### Number needed to switch (NNS)

The number of PID/SID patients needed to switch to reduce one nurse FTE was estimated by dividing the hours worked in a year (1,813) by the 49.2 hours in average annual savings in nurse time over three years (used to smooth out and take into account the increase in savings from 45.2 hours in year 1 to 51.2 annually thereafter).

### Population-wide savings potential

The population-wide savings potential in terms of nursing hours, FTEs and labor costs was estimated for Canada assuming 50% to 75% of currently treated patients are switched.

The prevalence of IgG treated PID/SID patients needed to be generated to anchor the population-wide savings potential since there are no population wide estimates of treated PID or SID (treated or untreated) patients in Canada. Martin et al. report there were 456 treated PID patients in BC in 2008/2009 with 4.46 million inhabitants at the time according to Statistics Canada. The prevalence of treated PID in BC (10.2/100,000) multiplied by the 2012 population from Statistics Canada was used to project the treated PID population to Canada.

The ratio of IgG issued between PID and SID patients was used to estimate the treated SID population. Utilization audits by the blood coordinating offices in the Atlantic Provinces (2011/2012) [13], British Columbia (2008/2009) [6], and Ontario (2007/2008) [14] provided the usage by indication ratios used to estimate the treated SID population. The IgG usage distributions for PID/SID among all indications were 17.4%/5.7% in the Atlantic Provinces, 26.1%/13.2% in BC, and 22.8%/12.9% in Ontario suggesting that approximately 65% of IgG usage is in PID and 35% in SID patients. The treated PID prevalence rate was used to estimate IgG treated SID patients in Canada.

### Sensitivity analyses

Univariate sensitivity analyses were performed to assess the robustness of the base-case results. The four parameters and the degree to which they were varied follows:

• Hours in Typical Work Year - decreased to 1,656 (36 hours × 46 weeks) to better reflect actual hours on the job given the number of national holidays in Canada and increased vacation time for experienced RNs

• Prevalence of Treated PID - changed to 12.4/100,000 (199 IgG treated patients) in the Atlantic Provinces (2011/2012) [13]

• Number of Saved Nursing Hours - increased to 157.5 to reflect saved nursing time from Ducruet et al. [10]; and, decreased IVIg nurse time by 25% to 128.7 hours over 3 years

• Nurse Compensation - changed bounded by the provinces with the highest and lowest maximum annual income for general duty RNs in Canada so that all provinces are contained within the range provided: compensation was adjusted upward by 13.4% to capture higher nurse compensation in Alberta and adjusted downward by 16.2% to capture lower nurse compensation in Quebec [15].

## Findings

### Net economic benefit

The expenditure in year 1 for nurse time to switch one patient to SCIg would be C$691 and C$345 annually thereafter to offset C$3,294 of hospital-based nursing time annually (Table 1). The net economic benefit per patient would be C$2,603 in year 1 and C$2,948 annually thereafter.

**Table 1:** Annual net economic benefit in Canada: Nurse time and compensation per patient switched from hospital-based IVIg to home-based SCIg

	**Year 1**	**Subsequent years**
**Home-based SCIg Therapy**		
Nurse hours: training	6	0
Nurse hours: monitoring	6	6
Nurse compensation, C$	691	345
**Hospital-based IVIg therapy**		
Nurse Hours: IV administration and monitoring	57.2	57.2
Nurse Compensation, C$	3,294	3,294
**Net economic benefit** (IVIg – SCIg)		
Savings: nurse hours	45.2	51.2
Savings: nurse compensation, C$	2,603	2,948

### Number needed to switch (NNS)

The number of patients that need to switch from IVIg to SCIg to recoup one nurse FTE is 36.85 (rounded to 37 for subsequent calculations). The net annual savings in nurse time would be 1,672.4 hours valued at C$96,297 in year 1 and 1,894.4 hours valued at C$109,080 annually thereafter (Table 2).

**Table 2:** Annual net economic benefit in Canada: Nurse time and compensation per 37 patients switched from hospital IVIg to home SCIg to recoup 1 nurse FTE

	**Year 1**	**Subsequent years**
**Home-based SCIg therapy**		
Nurse hours: training	222	0
Nurse hours: monitoring	222	222
Nurse compensation, C$	25,565	12,783
**Hospital-based IVIg therapy**		
Nurse hours: IV administration and monitoring	2,116.4	2,116.4
Nurse compensation, C$	121,862	121,862
**Net economic benefit** (IVIg – SCIg)		
Savings: nurse hours	1,672.4	1,894.4
Savings: nurse compensation, C$	96,297	109,080

### Population-wide savings potential

There are an estimated 5,486 patients with PID/SID (3,566 PID, 1,920 SID) receiving IVIg in Canada (Table 3). Switching 50% to 75% of IVIg-treated patients with PID/SID to SCIg has the potential to save, or free up, 223.3 to 335.1 nurse FTEs, representing C$23.3 to C$35.0 million in labor costs (Table 4).

**Table 3:** Estimated number of IgG-treated patients with PID and SID in Canada by province

**Province**	**2012 population (Thousands)**	**PID**	**SID**	**PID/SID combined**
Alberta	3,873.7	396.1	213.3	609.3
British Columbia	4,622.6	472.6	254.5	727.1
Quebec	8,054.8	823.6	443.5	1,267.1
Manitoba	1,267.0	129.5	69.8	199.3
New Brunswick	756.0	77.3	41.6	118.9
Newfoundland & Labrador	512.7	52.4	28.2	80.7
Nova Scotia	948.7	97.0	52.2	149.2
Ontario	13,505.9	1,380.9	743.6	2,124.5
Prince Edward Island	146.1	14.9	8.0	23.0
Saskatchewan	1,080.0	110.4	59.5	169.9
Territories	113.1	11.6	6.2	17.8
**Total**	34,880.6	3,566.0	1,920.0	5,486.0

**Table 4:** Estimated 3-year savings (Hours, FTEs, Costs) from switching 50% to 75% of treated patients with PID/SID from hospital IVIg to home SCIg in Canada by province

**Province**	**Savings with 50% switch rate**			**Savings with 75% switch rate**		
	**Hours**	**FTEs**	**Labor costs (C$ millions)**	**Hours**	**FTEs**	**Labor costs (C$ millions)**
Alberta	44,969	24.8	$ 2.59	67,453	37.2	$ 3.88
British Columbia	53,662	29.6	$ 3.09	80,493	44.4	$ 4.64
Quebec	93,506	51.6	$ 5.38	140,258	77.4	$ 8.08
Manitoba	14,708	8.1	$ 0.85	22,062	12.2	$ 1.27
New Brunswick	8,776	4.8	$ 0.51	13,164	7.3	$ 0.76
Newfoundland & Labrador	5,952	3.3	$ 0.34	8,928	4.9	$ 0.51
Nova Scotia	11,013	6.1	$ 0.63	16,520	9.1	$ 0.95
Ontario	156,786	86.5	$ 9.03	235,179	129.7	$ 13.54
Prince Edward Island	1,696	0.9	$0.10	2,544	1.2	$ 0.15
Saskatchewan	12,537	6.9	$ 0.72	18,806	10.4	$ 1.08
Territories	1,313	0.7	$ 0.08	1,969	1.1	$ 0.11
**Total**	404,918	223.3	$ 23.30	607,376	335.0	$ 34.98

### Sensitivity analyses

#### Hours in a typical work year

Changing the work-year hours to 1,656 decreases the number needed to switch to 33.66 to recapture one nurse FTE. The hourly cost of labor increases to C$63.04 as the number of hours in a work year decreases and annual wages and benefits remain constant. The expenditure in year 1 for nurse time to switch one patient to SCIg increases to C$756 and C$378 annually thereafter to offset C$3,606 in hospital-based nursing time annually.

#### Prevalence of treated PID

Increasing the prevalence of treated PID to 12.4/100,000 increases the number of treated PID/SID patients in Canada to 6,673. Switching 50% to 75% of IVIg patients to SCIg increases the potential savings to 271.7 to 407.5 nurse FTEs, representing C$28.4 to C$42.5 million in cost savings.

#### Number of saved nursing hours

Increasing saved nursing hours to 157.5 over 3 years would:

• Reduce number needed to switch to 34.53 to recoup one nurse FTE.

• Increase potential savings to 238.3 and 357.5 nurse FTEs, representing an increase in saved labor costs to C$24.9 and C$37.3 million assuming 50% to 75% of patients are switched.

Decreasing IVIg nurse time by 25% to 128.7 hours over 3 years would:

• Increase number needed to switch to 52.0 to recoup one nurse FTE.

• Decrease potential savings to 158.4 and 237.6 nurse FTEs, reducing saved labor costs to C$16.5 and C$24.8 million assuming 50% to 75% of patients are switched.

#### Nurse compensation

Increasing wages and benefits by 13.4% to C$118,390 (C$65.30 per hour) increases the net economic benefit to switch one SCIg patient in year 1 to C$2,952 and annually in subsequent years to C$3,343. Decreasing wages and benefits by 16.2% to C$87,487 (C$48.25 per hour) decreases the net economic benefit in year 1 to C$2,181 and in subsequent years to C$2,470 annually.

## Discussion

The paper presents three methods to express the economic benefits to payers in Canada from nurse time savings by shifting PID/SID patients from labor-intensive IVIg to less labor-intensive SCIg. We show that for every hour or Canadian dollar of nurse time in training and follow-up of SCIg patients in year 1 that 4.8 hours or C$ of nurse time for IVIg is offset. This increases to 9.5 hours or C$ annually thereafter. We estimate on a non-cumulative basis that for every 37 patients switched to SCIg, one nurse FTE is recouped. We show that given 5,486 PID/SID patients in Canada, between 223.3 to 335.0 FTEs could be redeployed to other service needs over a 3-year timeframe if 50% to 75% of IVIg patients were switched.

The 7-fold net increase in RN productivity observed in switching patients to SCIg far exceeds the one percent needed to reduce nurse shortages 50% by 2022 [1]. The projected annualized 3-year savings of 223 to 335 nurse FTEs represents 0.62% to 0.94% of the 11,900 FTEs in overtime for nurses in 2012 and 0.29% to 0.45% of the estimated 25,000-nurse shortage in 2013. This contribution would arise from a program that involves just 0.0157% of the Canadian population.

In the short term, nurse labor costs can be viewed as fixed and unlikely to vary with changes in patient volume. These costs, however, can be variable over the long run as nursing staff can be either downsized or upsized [16]. While opportunities to reallocate freed nursing time does not immediately reduce expenditures for hospitals, it has the potential to help manage the pressing demand for nurse time across the healthcare community in the short run.

The shift to SCIg in PID/SID patients likely has room for growth. The 2011/2012 IVIg and SCIg utilization for the Atlantic Provinces report that 13.5% of their PID patients have transitioned to self-administered home-based SCIg [13]. In the case of patients already switched, the initial investment has been made and the sustained benefits accruing. Considering that 69-81% of PID patients prefer SCIg to IVIg and 90-92% prefer home therapy, [8] there is ample opportunity for improved economic benefits to the health care system.

The following limitations are worth noting:

• The nurse time saved may be less than in our analysis. The 147.6 hours saved in our base-case is more conservative than the 157.5 hours reported in a pediatric PID population. We also did not take into account time saved for other IVIg-related institution staff (e.g., clerical aids, technicians), the patient, and family members.

• Patients switched to SCIg may want to switch back to IVIg and thus reduce the net economic benefit. It is unlikely that more than 5% of patients will switch back to IVIg given the preference data from Nicolay et al. [8].

• The prevalence data on treated PID from BC may not be generalizable across other provinces in Canada. The Ontario Intravenous Immune Globulin (IVIG) 2012 Audit [17] report indicates there are 509 PID patients receiving IVIg compared to our estimate of 1,381 patients: nearly a 3-fold difference. This may reflect a real difference in IVIg utilization between Ontario and the other provinces or the audit methodology.

• The proportion of treated PID/SID patients may vary from the 65%/35% split in IVIg issues by indication we used to estimate the prevalence of treated SID. We may have over/underestimated the prevalence of treated SID and by extension the population-wide savings potential of switching this population from IVIg to SCIg.

• Lastly, the salary-only data from the Canadian Federation of Nurses Unions in the sensitivity analysis does not reflect any variability there might be in benefits nor does it capture provisions for overtime pay.

The Canadian Institute for Health Information reports that compensation paid to health care providers is one of the most significant drivers of public sector health-care spending. Compensation constitutes 60% of the total hospital costs, and the single largest component of the workforce in hospitals is nurses [18]. Various Deputy Ministries of Health (MoH) within a province have a stake in ensuring efficient delivery of healthcare. An increase in nurse staff for SCIg may require additional funding separate from that for nurse staff devoted to IVIg. A better understanding across functional areas within the MoH of the offsets in nursing time by switching appropriate PID/SID patients from IVIg to SCIg may help advance a dialogue on the resources needed to facilitate these switches.

This paper seeks to clarify advantageous labor economics from a payer point of view. It did not attempt to conduct a full economic analysis of patients switching from IVIg to SCIg. Such analyses, if conducted, should benefit from this paper’s contributions.

## Conclusions

The shift from labor-intensive hospital-based IVIg to less labor-intensive home-based SCIg therapy has the potential to reduce overall health care costs borne by payers. In nations like Canada that have experienced chronic nursing shortages, the benefits of these patients switching modes of therapy would help alleviate nurse shortages. Health care professionals might consider advocating for home-based SCIg therapy for PID/SID patients when clinically appropriate.

## Abbreviations

BC: British Columbia; C$: Canadian dollars; CNA: Canadian nurses association; IgG: Immunoglobulin; FTE: Full-time equivalent; RN: Registered nurse; SCIg: Subcutaneous immunoglobulin; IVIg: Intravenous immunoglobulin.

## Competing interests

ASZ is employed by CSL Behring, which provides IVIg and SCIg formulations in Canada. WCG and SDB received remuneration for work on this project.

## Authors’ contributions

WCG conducted initial review to provide Canada specific context for understanding the importance of less nurse time with SCIg. All authors conceptualized approaches to estimate economic benefits of reduced nurse time to payers. WCG developed methods for, and conducted, analyses and drafted manuscript. SDB provided important clinical and Canadian healthcare perspectives in treating PID/SID with IgG. All authors interpreted data and approved the final version of this manuscript.

## Previous presentation

The results of this study were presented at the Annual Scientific Meeting of the Canadian Society of Allergy and Clinical Immunology; October 3–6, 2013; Toronto, ON, Canada.
